# Improving diagnosis in patients with obstetric antiphospholipid syndrome through the evaluation of non‐criteria antibodies

**DOI:** 10.1002/cti2.70021

**Published:** 2024-12-13

**Authors:** Daniel Álvarez, Hephzibah E Winter, Carlos J Velasquez Franco, Aleida Susana Castellanos Gutierrez, Núria Baños, Udo R Markert, Ángela P Cadavid, Diana M Morales‐Prieto

**Affiliations:** ^1^ Grupo Reproducción, Departamento Microbiología y Parasitología, Facultad de Medicina Universidad de Antioquia UdeA Medellín Colombia; ^2^ Placenta Lab, Department of Obstetrics Jena University Hospital Jena Germany; ^3^ Departamento de Reumatología Clínica Universitaria Bolivariana Medellín Colombia; ^4^ BCNatal Barcelona Center for Maternal‐Fetal and Neonatal Medicine (Hospital Clínic and Hospital Sant Joan de Déu), Institut Clínic de Ginecologia, Obstetrícia i Neonatologia Fetal i+D Fetal Medicine Research Center Barcelona Spain; ^5^ Grupo de Investigación en Trombosis, Departamento Medicina Interna, Facultad de Medicina Universidad de Antioquia UdeA Medellín Colombia

**Keywords:** antiphospholipid antibodies, antiphospholipid syndrome, autoimmunity, enzyme‐linked immunosorbent assay, pregnancy complication

## Abstract

**Objectives:**

Antiphospholipid syndrome (APS) is an autoimmune disease driven by antiphospholipid antibodies (aPL). Currently, APS diagnosis requires a combination of clinical manifestations (thrombosis and/or obstetric morbidity) and the persistent presence of at least one criteria aPL: anti‐cardiolipin antibodies (aCL), anti‐β2‐glycoprotein I antibodies (aβ2GPI) or lupus anticoagulant (LA). Patients with suggestive obstetric symptoms but lacking criteria aPL face diagnostic challenges. Non‐criteria aPL screening may enhance discrimination. This study proposes a classification incorporating both criteria and non‐criteria antibodies to improve obstetric APS diagnosis.

**Methods:**

Blood samples from non‐pregnant women (*n* = 68) with a history of vascular, obstetric, or vascular and obstetric manifestations were analysed. Among them, 30 had previous diagnosis of APS. Healthy women with proven gestational success were included as controls (*n* = 16). Criteria and non‐criteria (anti‐phosphatidylglycerol, anti‐phosphatidylethanolamine, anti‐phosphatidylinositol, anti‐phosphatidylserine and anti‐phosphatidic acid) IgG aPL were evaluated by ELISA and coagulation tests. Based on the resulting aPL profile, patients were reclassified. Responsiveness to treatment was obtained from medical records.

**Results:**

Criteria aPL levels marginally differentiated women previously managed as obstetric APS from unexplained/other causes of obstetric morbidity. Including non‐criteria aPL improved separation. The proposed classification identified an obstetric APS group that exhibits non‐criteria aPL and aβ2GPI titres below the cut‐off but higher than healthy women (7.88 vs. 2.47 SGU, *P* = 0.006). Compared to cases of other causes of obstetric morbidity, these patients retrospectively responded better to aspirin and/or heparin treatment (71.43% vs. 11.11%, *P* = 0.035).

**Conclusions:**

Assessing non‐criteria antibodies may identify isolated obstetric APS cases benefiting from established therapies.

## Graphical abstract

In this study, a classification system based on both criteria and non‐criteria antiphospholipid antibodies (aPL) is proposed to identify patients with Obstetric Antiphospholipid Syndrome. The resulting group exhibits non‐criteria aPL, and aβ2GPI titers below the cut‐off but higher than those in healthy women. These patients retrospectively responded better to aspirin and/or heparin treatment.

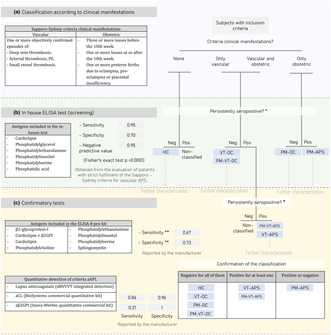

## Introduction

Antiphospholipid syndrome (APS) is an autoimmune disease and an acquired thrombophilia driven by a heterogeneous group of autoantibodies directed against phospholipids,^1^ phospholipid‐binding proteins and protein‐phospholipid complexes^2^ (antiphospholipid antibodies – aPL). Both *in vitro* and *in vivo* evidence,^2^, ^3^, ^4^, ^5^ along with the clinical characterisation of significant patient cohorts with APS,^6^ indicate the differentiation of this condition into two clinical entities that can manifest independently or concurrently: vascular and obstetric APS. Vascular APS is characterised by thrombotic events and it is associated with a non‐inflammatory hypercoagulable state that can occur even in males.^7^ Obstetric APS is distinguished by pregnancy complications associated with a local inflammatory process in the placenta that is accompanied by impaired trophoblast cell functions.^8^, ^9^ The presence of aPL and their deleterious effects in APS patients persists over time, so APS diagnosis is independent of pregnancy status.

Currently, no identified autoantibody profile is capable of predicting the onset of either vascular or obstetric APS. In contrast, there is evidence of monoclonal aPL with the capability to induce concomitantly pregnancy morbidity and vascular thrombosis in animal models.^10^ Seeking hypotheses to explain how the same group of autoantibodies can lead to two clinical entities with independent pathogenic mechanisms, Meroni *et al*.^11^ have proposed a ‘second hit’‐based hypothesis. The core elements of their hypothesis suggest that the differences between vascular and obstetric APS rely on the tissue distribution and aPL levels. In essence, β2‐glycoprotein‐I (β2GPI), the primary autoantigen in APS, localises primarily on trophoblast and endothelial cells of the decidua but not in other vascular tissues.^12^ Consequently, obstetric manifestations can occur even at low aPL levels. Non‐decidual endothelial cells, platelets and monocytes, critical components in vascular APS, are secondary targets for anti‐β2GPI (aβ2GPI) antibodies. Thus, a thrombotic event in vascular APS necessitates the sustained presence of high aPL titres and relies on a modification in the vascular binding of plasma β2GPI induced by a triggering factor or a ‘second hit’.^2^, ^7^, ^11^, ^12^, ^13^

Even though this hypothesis has robust experimental foundations, this tentative explanation of the differentiation between obstetric and vascular APS partially deviates from the classification criteria for APS diagnosis as set out in the Sapporo‐Sydney consensus,^14^ which the 2023 ACR/EULAR consensus has recently reaffirmed.^15^ According to these criteria, the persistent presence of moderate or high titres (titres > 99th percentile, at least in two occasions, at least 12 weeks apart) of aβ2GPI antibodies, anti‐cardiolipin (aCL) antibodies or lupus anticoagulant (LA) is a condition for APS diagnosis, both in its obstetric and vascular variants.^14^ However, different research groups have described that patients with classic obstetric clinical manifestations: (1) Are often intermittently seropositive for criteria aPL, (2) exhibit low titres of these antibodies, or (3) show persistent seropositivity for other autoantibodies that are not classically included in the diagnostic criteria for APS (e.g. aCL/vimentin complex antibodies, anti‐phosphatidylserine/prothrombin complex antibodies, anti‐phosphatidylinositol antibodies, anti‐phosphatidylcholine antibodies, anti‐sphingomyelin antibodies, among others).^16^, ^17^, ^18^, ^19^, ^20^ Patients with the aforementioned manifestations are managed as obstetric APS even if they do not fulfil the international classification criteria.^21^ In this context, some authors have proposed the term ‘seronegative’ obstetric APS.^16^, ^19^, ^21^ However, accepting this definition implies inherent challenges in differentiating patients with isolated – often ‘seronegative’ – obstetric APS from those experiencing pregnancy morbidity without known cause and those with known aetiology but without APS.

In this study, we aim to improve the classification of patients with obstetric morbidity to identify those associated with APS by using a panel of five non‐criteria aPL integrated into a high‐sensitivity in‐house test.

## Results and discussion

### The assessment of criteria aPL has marginal value for identifying patients with isolated obstetric APS

In the course of this research, 68 women were recruited and categorised as follows: healthy controls (*HC*; *n* = 16) with no significant pathological, surgical or pharmacological history and proven gestational success defined as at least one full‐term live birth without complications; patients with an isolated history of vascular thrombosis (*VT*) fulfilling Sapporo‐Sydney criteria diagnosis (*VT/APS*; *n* = 11) or diagnosed with other causes of vascular thrombosis (*VT/OC*; *n* = 11); patients with isolated history of pregnancy morbidity (*PM*), treated by their healthcare providers according to an APS diagnosis (*PM/APS*; *n* = 9), or by unexplained/other causes for PM (*PM/OC*; *n* = 11), and patients diagnosed according to the Sapporo‐Sydney criteria with a history of VT and PM (*PM‐VT/APS*; *n* = 10). These women were screened and reclassified according to their clinical manifestations and the results of a panel of criteria and non‐criteria aPL as described in the Methods section. To distinguish the original classification from the proposed new one, we use italic characters and replace the dash (‘‐’) with a slash (‘/’) in the abbreviations.

**Figure 1:**
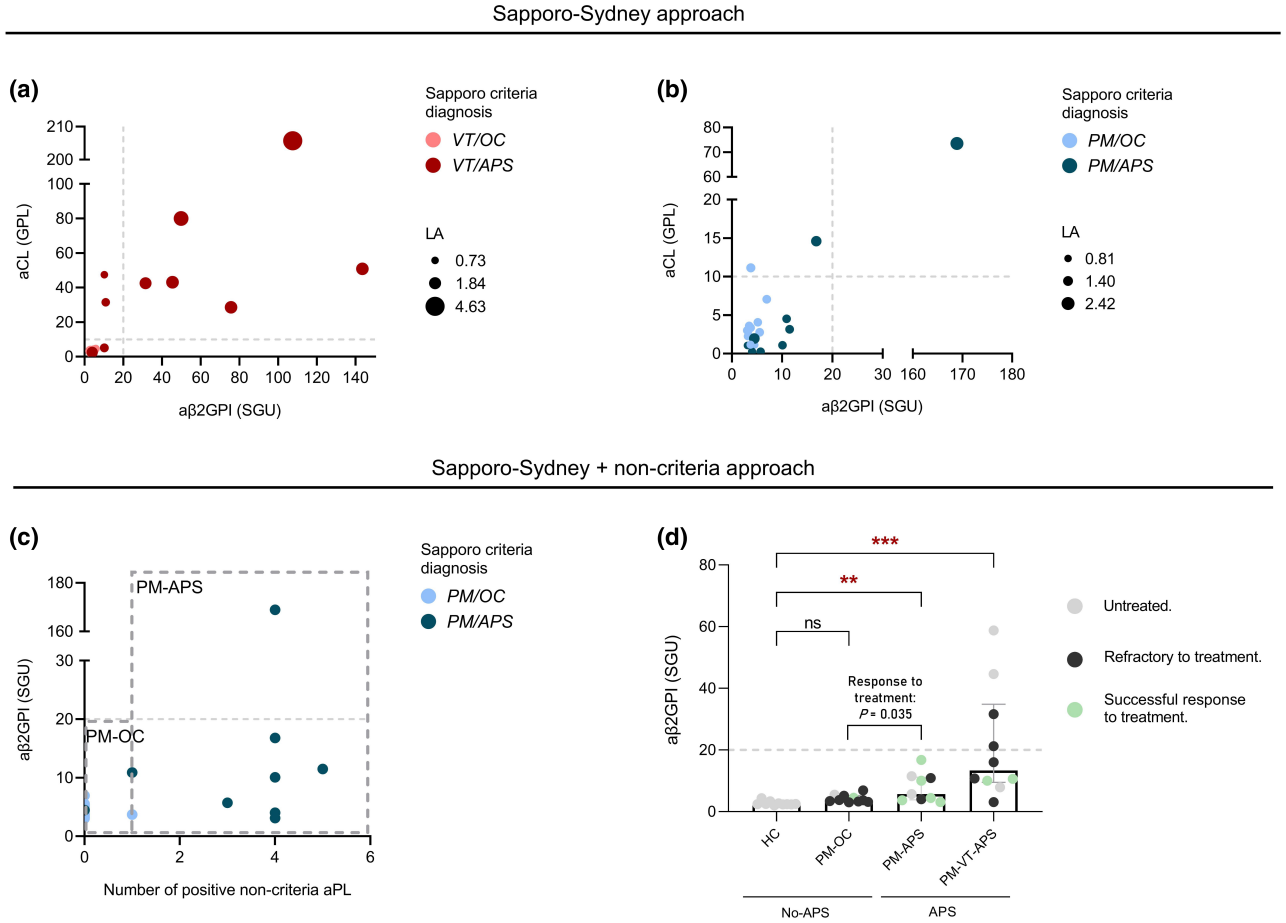
Classification based on both criteria and non‐criteria aPL improves the identification of isolated obstetric APS patients that benefit from established therapies. Patients previously diagnosed with APS following the classical Sapporo‐Sydney classification can be clearly distinguished from non‐APS subjects using only the criteria‐aPL profile in the presence of vascular **(a)** but not obstetric **(b)** manifestations. Women with direct oral anticoagulant consumption that makes testing for LA impractical are excluded from the analysis. Dotted lines represent the cut‐off points delimiting positive results according to Sapporo‐Sydney criteria. **(c)** As an alternative approach, patients with isolated obstetric manifestations were reclassified according to the persistent seropositivity for at least one non‐criteria aPL and categorised as PM‐APS or PM‐OC. **(d)** Titres of aβ2GPI differ in the newly classified groups (Kruskal–Wallis test, **P < 0. 01, ***P < 0.001), and retrospectively, PM‐APS had more often responded to standard APS treatment than PM‐OC (Success rate PM‐OC = 0.11, PM‐APS = 0.71, Fisher's exact test *P* = 0.035). An outlier value has been removed from the PM‐APS group. The analysis, both with or without this value, remains statistically significant. Please note that group names in italics represent diagnosis at the time of inclusion, while the names in regular font represent the diagnosis after the proposed classification. aCL, anti‐cardiolipin antibodies; aPL, antiphospholipid antibodies; APS, antiphospholipid syndrome; aβ2GPI, anti‐β2‐glycoprotein‐I; GPL, IgG phospholipid units; HC, healthy controls; PM, pregnancy morbidity; PM‐APS, obstetric APS; PM‐OC, obstetric manifestations other causes; PM‐VT‐APS, obstetric and vascular APS; SGU, standard units of aβ2GPI IgG; VT, vascular thrombosis.

The results from the tests widely employed in clinical and research settings for quantitative detection of criteria aPL (aβ2GPI, aCL and LA) confirm the usefulness of this method in differentiating between women with a history of vascular thrombosis related to APS and those with thrombosis caused by other factors. Consequently, patients with previous diagnoses of *VT/APS* (dark red dots) and those with *VT/OC* (light pink dots) were effectively distinguished solely based on the Sapporo criteria (Figure 1a). The proposed classification system confirmed the previous categorisation of VT patients, as 10 of 11 individuals previously classified as *VT/APS* remained in the VT‐APS group because of the seropositivity against at least one of the Sapporo criteria aPL (91% of *VT/APS* vs. 0% of *VT/OC*; *P* < 0.0001, sensitivity 0.91, specificity 1). Further, seven of 11 VT‐APS patients exhibited concomitantly positive results for at least two criteria aPL. VT‐APS patients showed 100% seropositivity against at least one of the tested non‐criteria aPL. Antibodies against phosphatidic acid (70%) and phosphatidylserine (60%) were the most common (Table 1 and Supplementary table 1).

**Table 1:** Description of the resulting groups from our systematic classification process

I. Study groups before the systematic classification process						
Groups	Control			APS		
	*HC* (*n* = 16)	*PM/OC* (*n* = 11)	*VT/OC* (*n* = 11)	*PM‐VT/APS* (*n* = 10)	*PM/APS* (*n* = 9)	*VT/APS* (*n* = 11)
Women persistently seropositive for anti‐phosphatidylglycerol (%)	25	6.3	0	70	78	45
Women persistently seropositive for anti‐phosphatidylethanolamine (%)	6.3	0	6.3	20	44	27
Women persistently seropositive for anti‐phosphatidylinositol (%)	13	0	0	40	56	36
Women persistently seropositive for anti‐phosphatidylserine (%)	19	0	0	60	78	55
Women persistently seropositive for anti‐phosphatidic acid (%)	6.3	0	0	50	56	64

II. Study groups after the systematic classification process (see Figure 2)						
*Non‐classified patients and controls*						
Healthy participants with successful pregnancies and positive results in at least one of the assessed aPL tests (*n* = 6)						
Women with a history of vascular thrombosis associated with causes other than APS, seropositive for the high‐sensitivity non‐criteria aPL test (*n* = 1)						
Patients with a previous diagnosis of vascular APS, without confirmation of the presence of criteria aPL (*n* = 1)						

	Control			APS		
Classified patients and controls	HC (*n* = 10)	PM‐OC (*n* = 10)	VT‐OC (*n* = 10)	PM‐VT‐APS (*n* = 10)	PM‐APS (*n* = 10)	VT‐APS (*n* = 10)
Age in years (median and interquartile range)	36 (33–37)	40 (36–42)	46 (32–49)	43 (37–51)	40 (37–43)	36 (30–46)
Vascular thrombosis‐related background						
Number of thrombosis events (median and range)	0	0	1 (1–3)	2 (1–10)	0	1.5 (1–4)
Women with DVT (%)	0	0	90	100	0	40
Women with PE (%)	0	0	40	40	0	70
Women with stroke (%)	0	0	0	10	0	20
Women with other arterial thrombosis (%)	0	0	0	0	0	10^a^
Women with OC use, HT, other risk factors for thrombosis or thrombophilia other than APS (%) – detailed list in Supplementary table 1	0	0	80^b^	30	0	20
Obstetric background						
Number of pregnancies (median and range)	2 (1, 2)	2.5 (2–5)	0 (0–4)	3.5 (1–7)	4* (2–7)	1 (0–4)
Number of live births (median and range)	2 (1, 2)	2 (0–3)	0 (0–3)	1 (0–3)	0 (0–3)	0.5 (0–3)
Number of gestational losses before 10 weeks (median and range)	0	0 (0–5)	0 (0–1)	1.5* (0–7)	1** (0–6)	0 (0–1)
Number of gestational losses at or after week 10 (median and range)	0	0 (0–1)	0	1 (0–2)	1** (0–3)	0
Women with preeclampsia, eclampsia, HELLP or IUGR (%)	0	60	0	60	30	10
Women with premature children (%)	0	60	10^c^	30	20	0
Number of pregnancies with complications other than gestational loss (median and range)	0	1^d^ ^,^** (0–2)	0^c^, ^e^ (0–1)	0.5 (0–2)	0 (0–2)	0^f^ (0–1)
Gestational success rate (median and interquartile range)^g^	1 (1)	0*** (0–0.23)	0.63 (0.1–0.9)	0*** (0–0.27)	0.09** (0–0.33)	0.75 (0–1)
ASA and LMWH response rate (%)^h^	NA	0.11	NA	0.29	0.71	NA
Another pathological history						
Women with hypothyroidism (%)	10	20	20	40	10	30
Women with neuropsychiatric disorders (%)	10	10	20	20	10	20
Women with other autoimmune diseases (%) – detailed list in Supplementary table 1	0	0	0	20	10	30
Pharmacological background at the time of sample collection						
Women receiving coumarin‐derived drugs (%)	0	0	10	60	0	70
Women receiving DOAC (%)	0	0	30	10	0	10
Women receiving LMWH (%)	0	0	0	0	0	0
Women receiving aspirin (%)	0	0	20	0	4	10
Women receiving chloroquine/hydroxychloroquine (%)	0	0	0	30	0	30
Laboratory results^i^						
INR (median and interquartile range)^j^	1.04 (0.93–1.16)	0.98 (0.92–1.01)	1.03 (0.84–1.21)	1.95 (1.19–2.36)	1.06 (0.89–1.43)	1.6 (1.18–2.53)
Lupus anticoagulant – NR (median and interquartile range) (positive > 1.2)^j^, ^k^	0.95 (0.81–1.08)	1.05 (0.93–1.09)	0.82 (0.80–0.88)	1.77^l^ ^,^** (1.45–1.95)	1.07 (0.97–1.58)	1.99^l^ ^,^* (1.25–2.40)
aCL antibodies in GPL (median and interquartile range) (positive > 10)	2.41 (1.68–3.52)	3.18 (2.49–4.81)	1.53 (1.01–3.37)	22.95^l^ ^,^* (9.70–164)	1.55 (0.85–7.03)	45.22^l^ ^,^* (30.7–77.3)
aβ2GPI antibodies in SGU (median and interquartile range) (positive > 20)	2.47 (2.35–2.88)	3.73 (3.24–5.23)	3.71 (3.26–5.92)	13.39*** (9.49–34.86)	7.88** (3.92–12.8)	47.62^l^ ^,^*** (10.7–113.9)
Women persistently seropositive for anti‐phosphatidylglycerol (%)	0	0	0	70	80	50
Women persistently seropositive for anti‐phosphatidylethanolamine (%)	0	0	0	20	50	20
Women persistently seropositive for anti‐phosphatidylinositol (%)	0	0	0	40	50	40
Women persistently seropositive for anti‐phosphatidylserine (%)	0	0	0	60	70	60
Women persistently seropositive for anti‐phosphatidic acid (%)	0	0	0	50	50	70

aCL, anti‐cardiolipin antibodies; APS, antiphospholipid syndrome; ASA, acetylsalicylic acid; aβ2GPI, anti‐β2‐glycoprotein‐I antibodies; DOAC, direct oral anticoagulants; HC, healthy women with proven gestational success; HT, hypertension; INR, international normalised prothrombin time range; IUGR, intrauterine growth restriction; LMWH, low‐molecular weight heparin; NR, normalised ratio; OC, oral contraceptives; PE, pulmonary embolism; PM‐APS, obstetric APS; PM‐OC, pregnancy morbidity related to causes other than the presence of aPL; PM‐VT‐APS, vascular and obstetric APS; VT‐APS, vascular APS; VT‐OC, vascular thrombosis related to causes other than the presence of aPL.

^a^ One patient with carotid artery thrombosis.

^b^ Includes two patients with homozygous factor II mutation.

^c^ One patient with deep venous thrombosis during pregnancy.

^d^ One patient with retroplacental hematoma.

^e^ One patient with gestational diabetes.

^f^ One patient had a stroke during pregnancy.

^g^ Ratio of pregnancies in each woman that progress without complications and result in a full‐term birth.

^h^ Ratio of women treated with LMWH and/or ASA who have at least one live full‐term birth documented while receiving drug treatment.

^i^ Obtained data from analysis of serum and plasma samples from each patient.

^j^ Excluded from these analyses are patients undergoing treatment with direct oral anticoagulants.

^k^ Assessed by clotting time in the presence of dilute Russell's viper venom, following ISTH recommendations.

^l^ Results above the established cut‐off point. Please note that group names in italics represent the diagnosis at the time of inclusion, while names in normal fonts represent the classification after re‐evaluation.

*P < 0.05, **P < 0.01, ***P < 0.001 (Kruskal–Wallis test, differences compared to HC group).

The current Sapporo‐Sydney criteria confirmed the obstetric APS diagnosis in only two of nine patients with pregnancy morbidity previously attributed to APS (*PM/APS*, dark blue dots) and failed to differentiate these from women with unexplained or non‐APS‐related PM (*PM/OC*, light blue dots) (Figure 1b).

As far as we know, extensive comparative analyses of the usefulness of criteria aPL levels for discrimination of patients with vascular and obstetric clinical manifestations of APS have not been attempted. Further, the discussion on diagnosis and treatment of ‘seronegative’ APS has focused on cohorts exhibiting only obstetric manifestations.^17^, ^22^ A large European retrospective study of a cohort of ‘seronegative’ APS patients reported more obstetric than vascular profiles.^23^ In this context, the clinical practice guidelines suggest that patients with manifestations of obstetric APS that do not strictly meet the definition of persistent seropositivity for Sapporo criteria aPL should be treated as if they meet the criteria (low‐dose aspirin and low‐molecular‐weight heparin).^21^ Consistent with the experience of our research group, there are publications documenting cases of women with seronegative APS who have experienced positive outcomes with this standard treatment.^16^, ^24^ However, the identification of this group remains challenging.

### Patients with pregnancy morbidity associated with APS can be distinguished through a highly sensitive test

Based on the positivity to Sapporo criteria and non‐criteria aPL, the proposed classification allows clear identification of a group of patients now designated PM‐APS (Figure 1c). The clinical records of these patients revealed that they had more often successfully responded to standard LMWH and/or ASA treatment previously, achieving at least one live birth at term (*P* = 0.035) (Figure 1d). Notably, although the aβ2GPI antibody titres of most patients in the PM‐APS group are below the cut‐off point of the Sapporo‐Sydney criteria (20 SGU), these values, unlike those of the PM‐OC group, are significantly higher than those from healthy women with proven gestational success (HC = 2.47 SGU, PM‐APS = 7.88 SGU, *P* = 0.006), reinforcing that this group can be differentiated and defined by significantly elevated aPL titres below the standard cut‐off (Table 1 and Figure 1d).

**Figure 2:**
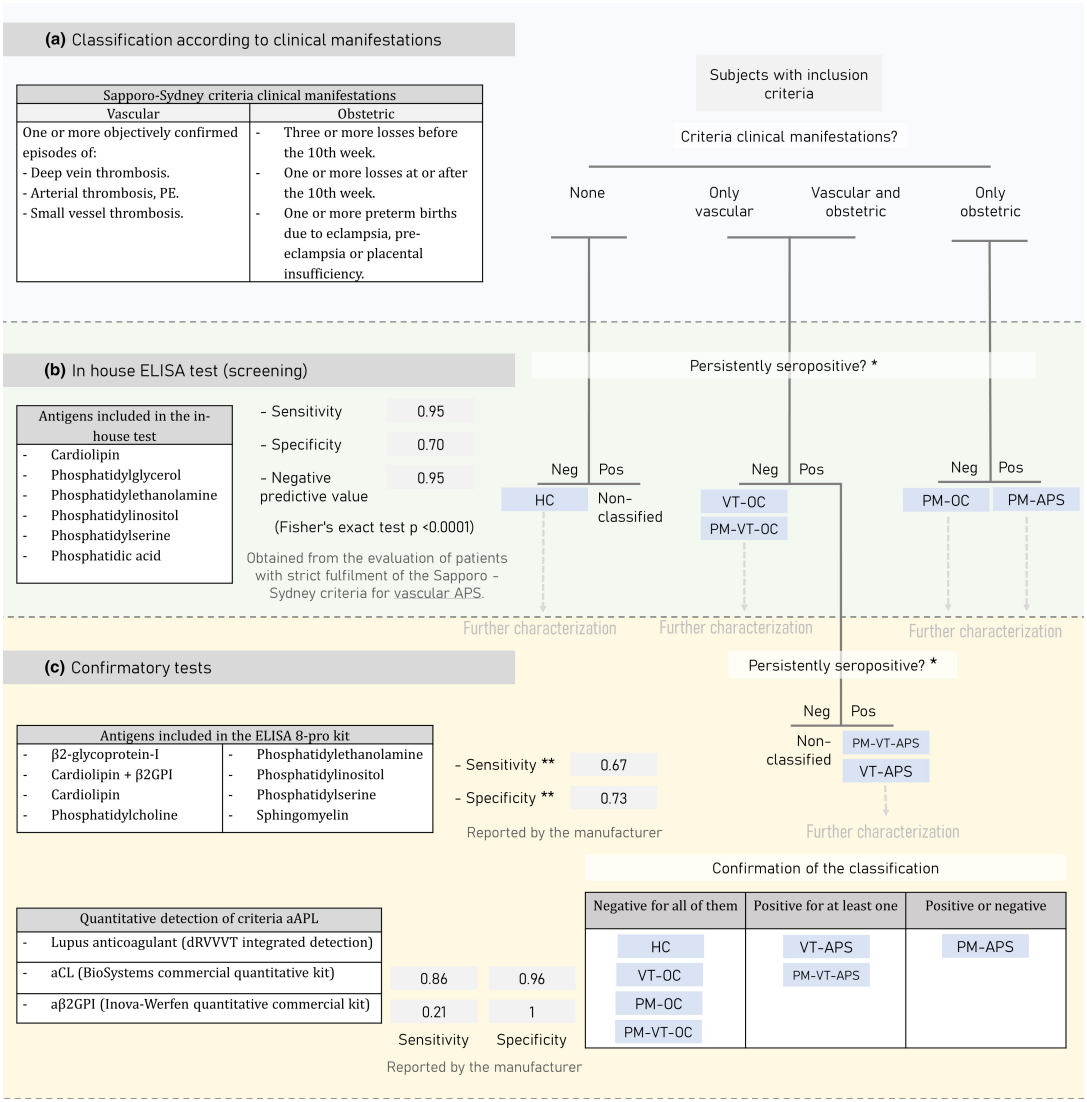
Proposed process for patient classification according to clinical manifestations and laboratory tests. Participants were classified according to three steps: **(a)** Based on the clinical manifestations described in the criteria for defining APS (Sapporo‐Sydney). **(b)** Screened using our reported high‐sensitivity in‐house ELISA test.^28^ **(c)** A semi‐quantitative ELISA test measuring both criteria and non‐criteria aPL was used for confirmation, followed by quantitative assessment of criteria aPL using widely used clinical tests. *Patients are considered persistently seropositive if they have two positive aPL tests at least 12 weeks apart. **Manufacturer‐reported values for aCL detection. aCL, anti‐cardiolipin antibodies; aPL, antiphospholipid antibodies; APS, antiphospholipid syndrome; aβ2GPI, anti‐β2‐glycoprotein‐I; dRVVT, diluted Russell's viper venom time; HC, healthy controls; PE, pulmonary embolism; PM‐APS, Obstetric APS; PM‐OC, obstetric manifestations other causes; PM‐VT‐APS, obstetric and vascular APS; PM‐VT‐OC, obstetric and vascular manifestations other causes; VT‐APS, vascular APS; VT‐OC, vascular manifestations other causes.

The alternative approach implements an in‐house ELISA test, which concomitantly assesses five non‐criteria aPL (Figure 2). In our hands, this test has proven to have a high sensitivity (0.95), a high negative predictive value (0.95) and a moderate specificity (0.70) when tested in samples from patients with a confirmed diagnosis of vascular APS (according to strict adherence to the modified Sapporo‐Sydney criteria; *n* = 20) and healthy women with proven gestational success (*n* = 16) or with a history of vascular thrombosis related to causes other than autoimmunity (*n* = 11) (Figure 2 and Supplementary figure 1). Other authors have described that the prevalence of positive results of similar non‐criteria aPL panels in seronegative APS patients can range between 18.8% and 36.8%. The prevalence of positive results in a cohort of criteria APS patients reached 83.2%.^20^, ^25^

All but two patients with PM‐APS were positive for at least two of the non‐criteria aPL. Anti‐phosphatidylglycerol antibodies (80%) were the most frequent, but scarce recent information can be found in the literature regarding their role in pregnancy. It should be noted that the analysis and comparison of the clinical data of PM‐APS and PM‐OC groups (age, number of pregnancies, gestational losses, premature births because of eclampsia, preeclampsia or placental insufficiency) does not allow a clear discrimination, as is possible with the use of non‐criteria aPL and the titres of aβ2GPI (Table 1).

Some cases remained unclassified when employing the proposed approach. Healthy women with proven gestational success showed seropositivity for the tested non‐criteria aPL (*n* = 6). These subjects could be considered as subclinical carriers of aPL. However, there is a lack of consensus about the clinical management of patients with positive aPL serology (even criteria aPL) who do not meet clinical criteria for APS.^26^ Likewise, two patients with a history of vascular thrombosis were negative for criteria but positive for non‐criteria aPL, remaining unclassified in our analysis. Whether these patients belong to a subtype of vascular APS remains unclear. This is challenging in the context of non‐criteria aPL, as their incidence in the general population and their association with increased risk of thrombosis or obstetric complications later in life remains to be established.

The described findings suggest that tests integrating non‐criteria aPL could be helpful in the classification of women with isolated clinical manifestations of obstetric APS. Furthermore, our results point to the differentiation of vascular and obstetric APS as variants distinguished by the low or intermittent aβ2GPI titres in the case of obstetric APS. Future prospective analyses of large patient cohorts may demonstrate the ability to discern between ‘actual seronegative’ patients who would not benefit from the proposed APS treatment and patients with isolated obstetric APS, often overlooked because of their low titres of criteria aPL.

## Methods

### Patient recruitment and sample collection

Adhering to the ethical principles established in the Helsinki Declaration, volunteers were recruited at the following centres: the Recurrent Abortion Program of Universidad de Antioquia; the Rheumatology Department of Clínica Universitaria Bolivariana; the Anticoagulation Clinic of San Vicente Fundación Hospital (all of the above located in Medellín, Colombia); the Jena University Hospital, Germany; and the Barcelona Centre for Maternal Fetal and Neonatal Medicine of the Hospital Clínic and Hospital Sant Joan de Déu, Barcelona, Spain. For all groups, vascular manifestations were considered to be at least one episode of clinically confirmed venous, arterial, or small vessel thrombosis requiring management with anticoagulant therapy. Patients with superficial venous thrombosis were excluded. Pregnancy morbidity was defined as at least one gestational loss at or after the tenth week of gestation, three or more gestational losses before the tenth week of gestation, or at least one case of preterm birth because of eclampsia, preeclampsia or placental insufficiency.

To avoid false‐positive results in aPL testing, pregnant women or women within the first 4 months postpartum and patients with cancer, as well as individuals with active or recent infections, acute clinical conditions or exacerbations of chronic diseases (major surgeries, major injuries or critical states including active or recent catastrophic APS) within the last 3 months were excluded.

Clinical data were recorded from each woman, and peripheral venous blood samples were collected to separate serum and platelet‐poor plasma. Standards were established to control pre‐analytical variables across the centres involved in sample collection. Serum and plasma samples were aliquoted and transported at −20°C, then stored at −80°C at the Grupo Reproducción Laboratory of Universidad de Antioquia, Colombia, until assessed using the aPL detection panel outlined in the following section.

### Antiphospholipid antibody profiling

The antiphospholipid antibody profile of each volunteer was assessed using the following test panel: (1) in‐house ELISA test: semi‐quantitative detection of IgG aCL, anti‐phosphatidylglycerol, anti‐phosphatidylethanolamine, anti‐phosphatidylinositol, anti‐phosphatidylserine and anti‐phosphatidic acid antibodies, previously standardised by our team (Grupo Reproducción, Medellín, Colombia)^27^, ^28^; (2) a semi‐quantitative solid phase enzyme immunoassay (AESKULISA Phospholipid‐8PRO‐GM; Aesku GmbH, Wendelsheim, Germany) for detection of IgG aCL, aβ2GPI, aCL/β2GPI, anti‐phosphatidylcholine, anti‐phosphatidylethanolamine, anti‐phosphatidylinositol, anti‐phosphatidylserine and anti‐sphingomyelin antibodies; (3) quantitative ELISAs for detection of IgG aCL (BioSystems, Barcelona, Spain) and IgG aβ2GPI (Quanta Lite β2 GPI IgG ELISA; Werfen, Barcelona, Spain). Additionally, LA antibodies were quantified by assessing clotting time in the presence of diluted Russell's viper venom (dRVVVT; Werfen) and in compliance with the recommendations established by the ISTH 2020 guideline.^29^

### Alternative patient classification and statistical analysis

Based on our autoantibody assessment, participants were re‐categorised through a systematic three‐step process into six new groups: Healthy controls (HC), Obstetric APS (PM‐APS), Vascular APS (VT‐APS), Obstetric and Vascular APS (PM‐VT‐APS), Vascular Thrombosis related to other causes (VT‐OC) and Obstetric Morbidity related to other causes (PM‐OC). An additional group of obstetric and vascular manifestations related to other causes (PM‐VT‐OC) was also considered but dismissed, as no patients were assigned to this group (Figure 2). Briefly, clinical features were used for initial classification in close compliance with the stated in the Sapporo‐Sydney criteria.^14^ Then, an initial screening for non‐criteria aPL was carried out using the semi‐quantitative in‐house ELISA tests, followed by a semi‐quantitative evaluation of criteria and non‐criteria aPL using commercial assays. Finally, a quantitative analysis of criteria aPL levels was done. In all cases, only persistently positive results (on two occasions at least 12 weeks apart) were considered true positive for classification into the APS groups. Generally, aPL tests provided by the medical records of the participants were used as the first result, and otherwise, two independent samples were assessed.

Women who had experienced a new episode of pregnancy‐related morbidity while being treated with heparin/low‐dose aspirin were, retrospectively, defined as ‘refractory to treatment’, and those who had a live birth at term were considered as of ‘successful response’.

The comparison of categorical variables was performed using Fisher's exact test. Compliance with the assumption of normality of data from quantitative variables was assessed using the Shapiro–Wilk test. Based on the results of this test, descriptive analyses were performed (mean and standard deviation, or median and interquartile range), and comparisons between groups were conducted using ANOVA or Kolmogorov–Smirnov tests. Dunn's or Dunnet's tests were used as *post hoc* tests. A ROC analysis was also performed. All statistical analyses were carried out using Prism GraphPad Software version 9.0.0.

## Conflict of interest

The authors declare no conflict of interest.

## Author contributions

**Daniel Álvarez:** Conceptualization; data curation; formal analysis; investigation; methodology; visualization; writing – original draft. **Hephzibah E Winter:** Investigation; writing – review and editing. **Carlos J Velasquez Franco:** Investigation; writing – review and editing. **Aleida Susana Castellanos Gutierrez:** Investigation. **Núria Baños:** Investigation. **Udo R Markert:** Funding acquisition; project administration; resources; supervision; writing – review and editing. **Ángela P Cadavid:** Funding acquisition; project administration; resources; supervision; writing – review and editing. **Diana M Morales‐Prieto:** Conceptualization; funding acquisition; project administration; resources; supervision; writing – original draft; writing – review and editing.

## Ethics statement

The authors confirm the adherence of this work to the Helsinki Declaration on ethical principles for medical research involving human subjects. This project was reviewed and approved by the following Ethics Committees: Comité de Ética del Instituto de Investigaciones Médicas, Universidad de Antioquia, Colombia (No. 019‐2020 and No. 004‐2017); The responsible internal UKJ Ethics Committee (Reg‐Nr. 2020‐2022—Material); Comité de Ética de la Investigación con medicamentos del Hospital Clínic de Barcelona HCB/2022/0265.

## Supporting information


Supplementary figure 1
Supplementary table 1

## Data Availability

The data that support the findings of this study are available from the corresponding author upon reasonable request.
