# Supplementary material for: Improving diagnosis in patients with obstetric antiphospholipid syndrome through the evaluation of non‐criteria antibodies
Source: Clin Transl Immunology. 2024 Dec 13;13(12):e70021. doi: 10.1002/cti2.70021 (PMC11638733; doi:10.1002/cti2.70021)
Supplement: Supplementary file 1 — Supplementary figure 1 Supplementary table 1 [file CTI2-13-e70021-s001.docx]

**Short Communication**

**Improving diagnosis in patients with obstetric antiphospholipid syndrome through the evaluation of non-criteria antibodies**

Daniel Álvarez, Hephzibah E Winter, Carlos J Velasquez Franco, Aleida Susana Castellanos Gutierrez, Núria Baños, Udo R Markert, Ángela P Cadavid, Diana M Morales-Prieto

**Supporting information**

**Supplementary table 1.** Additional clinical data

| Groups | Control | | | APS | | |  |
| --- | --- | --- | --- | --- | --- | --- | --- |
|  | HC | PM-OC | VT-OC | PM-VT-APS | PM-APS | VT-APS |  |
|  | (n=10) | (n=10) | (n=10) | (n=10) | (n=10) | (n=10) |  |
| Women with risk factors for thrombosis or thrombophilia other than APS (%) | 0 | 0 | 80 | 30 | 0 | 20 |  |
|  |  |  |  |  |  |  |  |
| Women with sticky platelet syndrome (%) | 0 | 0 | 10 | 0 | 0 | 0 |  |
|  |  |  |  |  |  |  |  |
| Women with proven Prothrombin G20210A mutation (%) | 0 | 0 | 20 | 0 | 0 | 0 |  |
|  |  |  |  |  |  |  |  |
| Women with persistent elevation of FVIII (%) | 0 | 0 | 10 | 0 | 0 | 0 |  |
|  |  |  |  |  |  |  |  |
| Oral contraceptive intake (%) | 0 | 0 | 30 | 0 | 0 | 0 |  |
|  |  |  |  |  |  |  |  |
| Hypertension (%) | 0 | 0 | 10 | 20 | 0 | 20 |  |
|  |  |  |  |  |  |  |  |
| Thrombosis post-surgery (%) | 0 | 0 | 10 | 0 | 0 | 0 |  |
|  |  |  |  |  |  |  |  |
| Atrial fibrilation (%) | 0 | 0 | 0 | 10 | 0 | 0 |  |
|  |  |  |  |  |  |  |  |
| Smoker (%) | 0 | 0 | 0 | 10 | 0 | 0 |  |
|  |  |  |  |  |  |  |  |
| Obesity (%) | 0 | 0 | 0 | 0 | 0 | 0 |  |
|  |  |  |  |  |  |  |  |
| Women with other autoimmune diseases (%) | 0 | 0 | 0 | 20 | 10 | 30 |  |
|  |  |  |  |  |  |  |  |
| SLE (%) | 0 | 0 | 0 | 10***** | 0 | 20****** |  |
|  |  |  |  |  |  |  |  |
| Multiple sclerosis (%) | 0 | 0 | 0 | 10 | 0 | 0 |  |
|  |  |  |  |  |  |  |  |
| Hashimoto's disease (%) | 0 | 0 | 0 | 0 | 10 | 0 |  |
|  |  |  |  |  |  |  |  |
| Rheumatoid arthritis (%) | 0 | 0 | 0 | 0 | 0 | 10******* |  |
|  |  |  |  |  |  |  |  |

***** One patient with antinuclear antibodies 1:320

****** One patient with antinuclear antibodies 1:160 and one patient with ANA 1:164.

******* One patient with antinuclear antibodies 1:80 and low C3 protein.


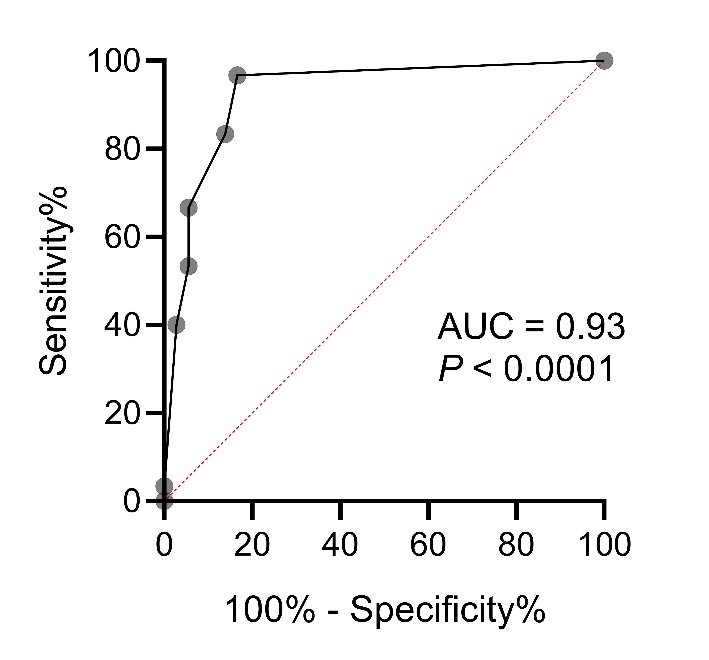


**Supplementary figure 1.** Comparison of sensitivity and the reciprocal of specificity according to the number of non-criteria aPL of the high-sensitivity in-house test for which controls and patients with a previous diagnosis of APS are positive (ROC curve).
